# Structural and Enzymatic Characterization of the Phosphotriesterase OPHC2 from *Pseudomonas pseudoalcaligenes*

**DOI:** 10.1371/journal.pone.0077995

**Published:** 2013-11-04

**Authors:** Guillaume Gotthard, Julien Hiblot, Daniel Gonzalez, Mikael Elias, Eric Chabriere

**Affiliations:** 1 URMITE UMR CNRS-IRD 6236, IFR48, Faculté de Médecine et de Pharmacie, Université de la Méditerranée, Marseille, France; 2 Weizmann Institute of Science, Biological Chemistry, Rehovot, Israel; University of Cambridge, United Kingdom

## Abstract

**Background:**

Organophosphates (OPs) are neurotoxic compounds for which current methods of elimination are unsatisfactory; thus bio-remediation is considered as a promising alternative. Here we provide the structural and enzymatic characterization of the recently identified enzyme isolated from *Pseudomonas pseudoalcaligenes* dubbed OPHC2. OPHC2 belongs to the metallo-β-lactamase superfamily and exhibits an unusual thermal resistance and some OP degrading abilities.

**Principal findings:**

The X-ray structure of OPHC2 has been solved at 2.1 Å resolution. The enzyme is roughly globular exhibiting a αβ/βα topology typical of the metallo-β-lactamase superfamily. Several structural determinants, such as an extended dimerization surface and an intramolecular disulfide bridge, common features in thermostable enzymes, are consistent with its high T_m_ (97.8°C). Additionally, we provide the enzymatic characterization of OPHC2 against a wide range of OPs, esters and lactones.

**Significance:**

OPHC2 possesses a broad substrate activity spectrum, since it hydrolyzes various phosphotriesters, esters, and a lactone. Because of its organophosphorus hydrolase activity, and given its intrinsic thermostability, OPHC2 is an interesting candidate for the development of an OPs bio-decontaminant. Its X-ray structure shed light on its active site, and provides key information for the understanding of the substrate binding mode and catalysis.

## Introduction

**Figure 1:**
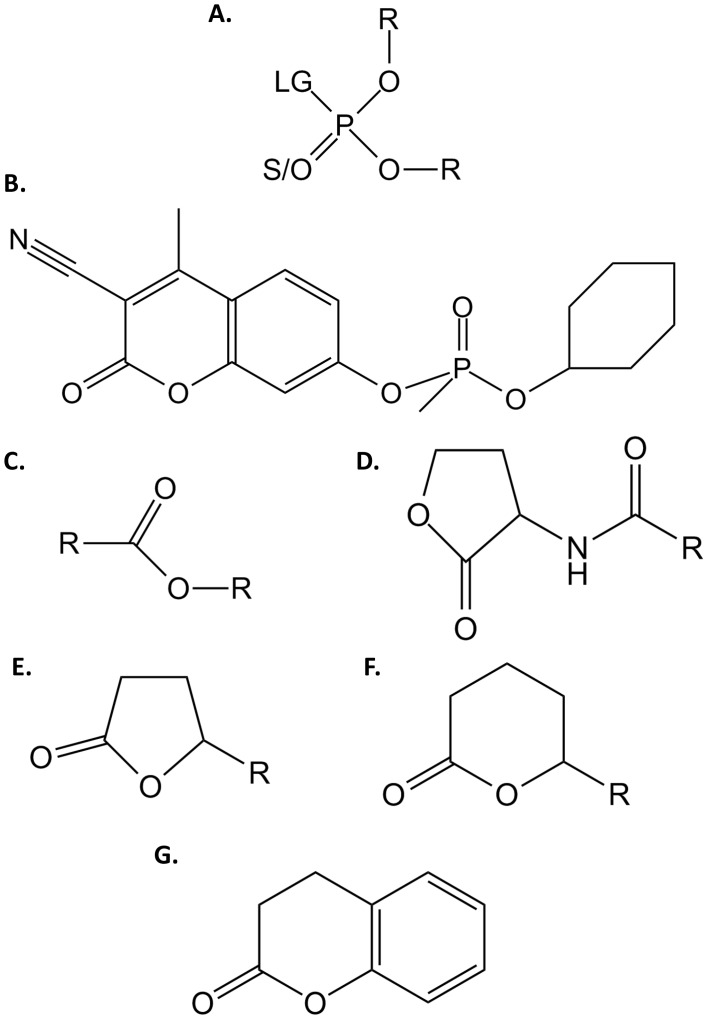
Chemical structure of tested substrates. Chemical structures of (**A.**) phosphotriesters, (**B.**) CMP-coumarin, (**C.**) esters, (**D.**) Acyl-Homoserine Lactones, (**E.**) γ-lactones, (**F.**) δ-lactones and (**G.**) dihydrocoumarin are presented. For phosphotriesters, R corresponds to different nature of substituents; LG corresponds to the leaving group which can be F, S-R, O-R or CN. The terminal substituent could be S atom if the molecule is a thionophosphotriester or an O atom if the molecule is an oxonophosphotriester. For esters, R corresponds to different nature of substituent. For AHLs and γ/δ-lactones, R corresponds to different size of acyl chain.

Organophosphates (OPs; **Fig. 1A**) are well known neurotoxic compounds which irreversibly inhibit the acetylcholinesterase, a key enzyme in the nerve signal transmission [1]. OPs are widely used as agricultural insecticides [2] and their most toxic representatives have been developed as chemical warfare agents (*e.g.* tabun, sarin, soman or VX) [3]. These compounds are still massively used as pesticides resulting in considerable pollutions [4], [5]. Current methods for removing them are slow, cost prohibitive [6], and generate secondary pollution. Novel methods of remediation such as enzyme-mediated decontamination, are therefore highly desirable and under intensive research [7], [8].

OPs pesticides have been massively used since the 1950’s, leading to the fast emergence of microorganisms that are capable of degrading OPs, and that can probably utilize them as carbon and phosphorus source [9]. Several OrganoPhosphorus Hydrolases (OPHs) have been identified so far, belonging to different protein families: the OrganoPhosphorus Acid Anhydrolases (OPAAs; EC 3.4.13.9) related to the prolidases [10], [11], the paraoxonases (PONs; EC 3.1.8.1) [12], the PhosphoTriEsterases (PTEs; EC 3.1.8.1) and the Phosphotriesterase-Like Lactonases (PLLs; EC 3.1.1.81) from the amidohydrolase superfamily, and finally the OPHs from the metallo-β-lactamase superfamily [13]. PTEs, isolated from *Brevundimonas diminuta* [14] and *Agrobacterium radiobacter* [15], are the most characterized OPHs so far and exhibit near diffusion-limit rate for the insecticide paraoxon as substrate [14]. PTEs are believed to have emerged from native lactonases with promiscuous phosphotriesterase activity such as the PLLs [16]–[18].

A recently identified OPH, named OPHC2 (GenBank ID: AJ605330), has been isolated from *Pseudomonas pseudoalcaligenes* [19], [20] but also from *Stenotrophomonas sp.* SMSP-1 (98% identity) [21]. This enzyme shares about 45% sequence identity with the Methyl-Parathion Hydrolases (MPHs; EC 3.1.8.1). MPHs enzymes are isolated from several organisms, such as *Pseudomonas putida*, *Pleisiomonas sp*. M6, *Ochrobactrum sp.* M231 or *Pseudomonas sp.* WBC3, and hydrolyze methyl-parathion with high efficiency (*i.e.* k_cat_/K_M_∼10^6^ M^−1^ s^−1^) [22]. The structure of MPH from *Pseudomonas sp.* WBC3 was solved and revealed an αβ/βα sandwich fold typical of the metallo-β-lactamase superfamily, forming a homodimer [22]. Containing a Zn(II) bi-metallic catalytic site bridged by a water molecule, the MPHs catalytic mechanism is presumed to be similar to that of other OPHs [22]. The bridging catalytic water molecule is activated by the bi-metallic active site and serves as the nucleophile that attacks the phosphorus center of the bound substrate *via* a SN_2_ mechanism [22].

OPHC2 has been previously shown to exhibit methyl-parathion hydrolyzing activity [19]. Although the enzyme originates from a mesophilic soil bacterium [23], its optimum temperature for catalysis is reported to be 66°C. In this article, we report the biochemical, enzymatic and structural analysis of OPHC2 from *P. pseudoalcaligenes*. In combination, these results allows us to propose possible explanations for the thermostability of OPHC2 and its substrate preference.

## Materials and Methods

### Sequence Alignment

The alignments were performed using the *T-coffee* server (expresso) [24], [25], and subsequently manually improved. The phylogenetic tree was performed using *PhyML* [26] and default parameters. The sequence alignment was drawn with *BioEdit* 7.1.3. Protein sequence identities were calculated using *ClustalW* server [27].

### Protein Production and Purification

The protein production and purification was performed as previously explained [20]. Briefly, the protein production was performed in *E. coli* strain BL21(DE_3_)-pGro7/GroEL (TaKaRa). Purification was performed using a previously described procedure [18], [28] that takes advantage of the high stability of the target protein by performing a heating step of 30 minutes at 70°C followed by a differential ammonium sulfate precipitation to eliminate remaining thermostable proteins. The sample is subsequently loaded on a size exclusion column [20]. Proteins were quantified using a nanospectrophotometer (nanodrop, thermofisher scientific, France) with the protein molar extinction coefficient (ε_280 nm_ = 38 390 M^−1^ cm^−1^) calculated by the *PROT-PARAM* server [29].

### Determination of the Oligomerization State

Oligmerization state determination was performed using a size exclusion column S75 10/300 GL (GE-Healthcare) calibrated with the Gel Filtration Low Molecular Weight calibration kit (GE-Healthcare) in *activity buffer* (50 mM HEPES pH 8, 150 mM NaCl, 0.2 mM CoCl_2_). 145 µg of OPHC2 enzyme was separated using a flow rate of 0.5 ml.min^−1^ on an ÄKTA avant chromatography apparatus (GE-Healthcare) running with the *UNICORN 6.1* software. Dynamic light scattering (DLS) experiments were performed at room temperature using zetasizer nano series apparatus (Malvern, UK) and the Zetasizer software. 30 µL of purified OPHC2 (5 mg.ml^−1^) was used in the *activity buffer* to measure the hydrodynamic radius of particles at 633 nm allowing estimation of a theoretical enzyme molecular weight.

### Kinetic Characterization

Catalytic parameters were evaluated at 25°C, and recorded with a microplate reader (Synergy HT, BioTek, USA) and the Gen5.1 software in a 6.2 mm path length cell for 200 µL reaction in 96-well plate as previously explained [18]. Catalytic parameters were obtained by fitting the data to the Michaelis-Menten (MM) equation [30] using *Graph-Pad Prism 5* software. When V_max_ could not be reached in the experiments, the catalytic efficiency was obtained by fitting the linear part of MM plot to a linear regression using *Graph-Pad Prism 5* software.

Standard assays were performed in *activity buffer* by measuring the time course hydrolysis of *p*NP derivatives (ε_405 nm_ = 17 000 M^−1^ cm^−1^) of OPs (**Fig. 1A**), esters (**Fig. 1C**). For malathion (**Fig. S1V**), 2 mM DTNB was added to the buffer (ε_412 nm_ = 13 700 M^−1^ cm^−1^). The time course hydrolysis of phenyl-acetate (**Fig. S1VI**) and dihydrocoumarin (**Fig. 1G**) were monitored at 270 nm (ε_270 nm_ = 1 400 M^−1^ cm^−1^) and at 412 nm for coumarin nerve agent derivative of cyclosarin (CMP-coumarin **Fig. 1B**; ε_412 nm_ = 37 000 M^−1^ cm^−1^). The kinetics for the lactonase activities were performed using a previously described protocol [18]. The time course hydrolysis of lactones were performed in *lactonase* buffer (2.5 mM Bicine pH 8.3, 150 mM NaCl, 0.2 mM CoCl_2_, 0.25 mM Cresol purple and 0.5% DMSO) with different AHLs (**Fig. 1D**) [*i.e.* C4-AHL (*r*), 1 mM; C6-AHL (r), 2 mM; 3-oxo-C6-AHL (*l*), 2 mM; C8-AHL (*r*), 1 mM; 3-oxo-C8-AHL (*l*), 2 mM; 3-oxo-C10-AHL (*l*), 2 mM] (**Fig. S1IX–XIV**) and oxo-lactones (**Fig. 1EF**) [*i.e.* ε-caprolactone, 5 mM; γ-heptanolide (*r*), 5 mM; Nonanoic-γ-lactone (*r*), 5 mM; Nonanoic-δ-lactone (*r*), 5 mM; Undecanoic-γ-lactone (*r*), 5 mM] (**Fig. S1XV–XIX**). Cresol purple (pK_a_ 8.3 at 25°C) is a pH indicator (577 nm) used to follow the lactone ring hydrolysis that cause an acidification of the medium.

### Thermostability Analysis

Circular Dichroïsm (CD) spectra were recorded as previously described [18] using Jasco J-810 spectropolarimeter equipped with Pelletier type temperature control system (Jasco PTC-4235) in 1 mm thick quartz cell and using the *Spectra Manager* software. To determine the melting temperature of the protein, the denaturation was recorded at 222 nm by increasing the temperature from 20 to 95°C (at 2°C/min) in 10 mM sodium phosphate buffer at pH 8 containing increasing concentrations (1–3 M) of guanidinium chloride. The theoretical T_m_ without guanidinium chloride was extrapolated at the y-intercept by a linear fit using the *Graph-Pad Prism 5* software.

### Crystallization

Crystallization was performed as previously published [20]. Briefly, crystals were obtained by the sitting drop vapour diffusion method set up in a 96-well plate. Crystals grow reproducibly after three months at 293 K in drops (2∶1 and 1∶1 protein:reservoir ratio) of condition 1 (10% PEG 8000, 100 mM Tris-HCl pH 7 and 200 mM MgCl_2_) and condition 2 (10% PEG 3000, 100 mM Sodium cacodylate pH 6.5 and 200 mM MgCl_2_).

### Data Collection and Structure Refinement

Crystals were transferred into a cryo-protectant solution composed of the reservoir solution and 20% (v/v) glycerol prior to flash-cooling in liquid nitrogen. X-ray diffraction dataset was collected at 100 K using synchrotron radiation at the Proxima-1 beam line (SOLEIL, Gif-sur-Yvette, France) and a PILATUS-6M detector (DECTRIS, Switzerland). X-ray diffraction data were integrated and scaled with the *XDS* package [31] (**Table 1**). The phases were obtained by molecular replacement with *PHASER* [32] as previously described, using the MPH structure as a starting model (PDB ID 1P9E) [20]. The model was subsequently built with *Coot* [33] and refined using *REFMAC5* [34] and *PHENIX* [35]. A total of five monomers (two dimers and one monomer of a symmetry related dimer) were found per asymmetric unit. One of these dimers is highly agitated in the crystal, resulting in a poor electron density. The model and structure factors were deposited under the Protein Data Bank (PDB) code 4LE6.

**Table 1:** Data collection and refinement statistics of OPHC2 structures.

Data collection	
PDB ID	4LE6
Beamline	PROXIMA-1
Wavelength (Å)	0.980
Detector	PILATUS 6M
Oscillation (°)	0.15
Number of frames	1200
Resolution (Å) (last bin)	2.1 (2.2–2.1)
Space group	C2
Unit-cell parameters (Å)	a = 109.9, b = 63.8,c = 221.3, β = 101.8
No. of observed reflections (last bin)	252270 (24317)
No. of unique reflections (last bin)	82530 (9469)
Completeness (%)(last bin)	93.7 (82.6)
R_meas_ (%) (last bin)	6.5 (50.1)
I/σ(I) (last bin)	13.67 (3.07)
Redundancy (last bin)	3.06 (2.57)
Mosaicity (°)	0.508
Refinement statistics	
Rfree/Rwork	20.99/17.32
No. of total model atoms	19946
Ramachandran favored	93.5
Ramachandran outliers	0.8
Generously allowed rotamers	5.7
Rmsd from ideal	
Bond lengths (Å)	0.008
Bond angles (°)	1.111

### Structural Modeling

The region 168 to 210, lacking from OPHC2 structure, was modelled using *CHIMERA* [36] and *Modeller 9.11* [37] with the structure of MPH as template.

### Anomalous X-ray Scattering Experiments

The chemical nature of the bi-metallic center was studied using anomalous X-ray fluorescence. Two datasets were collected consisting of 3600×0.1° at 2.6 and 3.1 Å resolution at respective wavelengths lower (1.2835 Å) and higher (1.2822 Å) than the Zn-K absorption edge. Moreover, the X-ray fluorescence spectrum of OPHC2 crystal has been collected.

### Structure Analysis

Structural comparisons were made using the crystal structures of MPH (PDB ID 1P9E) and AiiA (PDB ID 2A7M). Structure illustrations, analysis and comparisons were performed using *PyMOL* [38]. Vacuum electrostatic potentials and surface representation were computed under *PyMOL* using a solvent probe of 1.4 Å radius. The surface of the dimer interface and the number of hydrogen bonds and salt bridges were computed using *PISA* [39] available from *PDBe* web interface [40]. Root mean square deviations (RMSD) were calculated on α-carbon using the *align* command under *PyMOL* interface [38].

## Results

**Figure 2:**
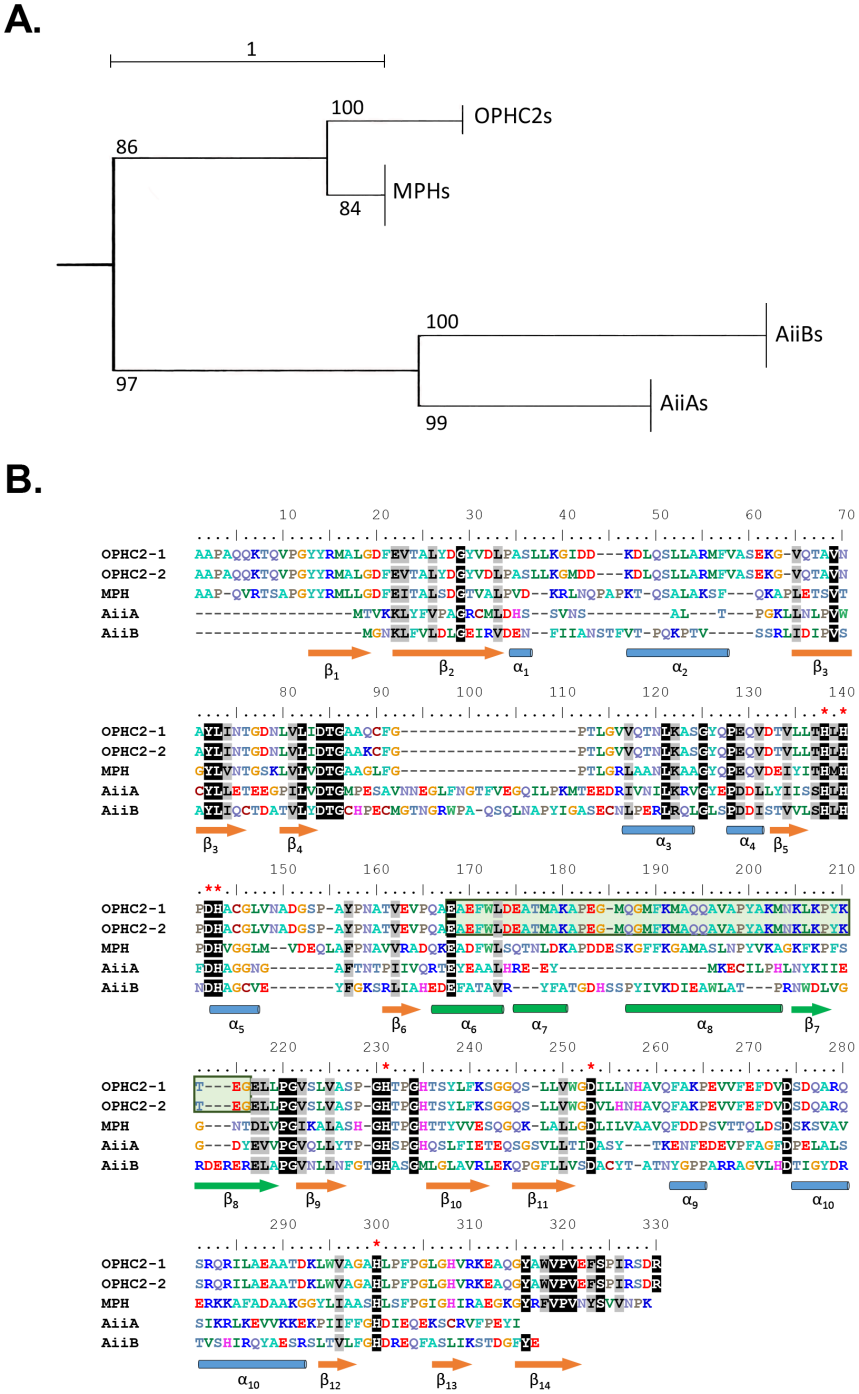
Phylogenetic analysis of OPHC2 enzymes. **A**. Simplified phylogenetic tree of several OPHs (OPHC2s and MPHs) and lactonases (AiiAs and AiiBs) exhibiting a αβ/βα topology. Sequences were selected from NCBI blast (sequence identity>40%) using input query of OPHC2 from *P. pseudoalcaligenes*, MPH from *Stenotrophomonas* sp. Dsp-4, AiiA from *B. thuringiensis* and AiiB from *A. fabrum* str. C58. Alignment was performed using the *T-coffee* server and the tree was built using *PhyML*. The tree has been arbitrarily rooted for clarity. Bootstraps values are indicated. **B**. Sequence alignment of OPHC2 from *P. pseudoalcaligenes* (OPHC2-1) and *Stenotrophomonas* sp. SMSP-1 (OPHC2-2), MPH from *Stenotrophomonas* sp. Dsp-4, AiiA from *B. thuringiensis* and AiiB from *A. fabrum* str. C58. Conserved residues are represented with black font while similar residues are represented with grey font. Secondary structures are annotated according to OPHC2 structure (**Fig. 3C & S4) (**β-sheets are represented by orange arrows and α-helices are represented by light blue tubes). Conserved residues involved in the divalent cations coordination are indicated by red stars. Modeled parts of OPHC2 are represented in green (see also **Fig. S4**). This lacking part is also highlighted by a green font.

OPHC2 belongs to the metallo-β-lactamase superfamily and shares significant homology with other representatives such as MPH (identity = 48%; similarity = 58.6%), and lower sequence identity with the lactonases AiiA (17–18%) and AiiB (14%) (**Table S1**) [41]. A phylogenetic tree (**Fig. 2A**) clearly shows that AiiAs and AiiBs in the one hand, MPHs and OPHC2s on the other, form different clades. A sequence alignment between OPHC2, MPH, AiiA and AiiB sequences (**Fig. 2B**) highlights the common HXHXDH motif typical of the bi-metallic catalytic center of the metallo-β-lactamase superfamily [42]. The main variable regions are concentrated in the helix/loop parts of the enzymes.

### Biochemical Characterization

Size exclusion chromatography experiments performed on OPHC2 revealed an apparent molecular weight of 58.3 kDa corresponding to an intermediate size between monomeric (32 kDa) and dimeric (64 kDa) forms (**Fig. S2A**). This discrepancy may be due to a slightly anomalous Stokes radius relative to its actual mass, or may reflect a rapid equilibrium between monomeric and dimeric forms of the enzyme as previously proposed [43]. DLS experiments revealed an apparent size of 74.9±11.3 kDa (**Fig. S2B**), suggesting that OPHC2 is a dimer in solution. OPHC2 melting temperature (T_m_) was also determined to 97.8±3.2°C by circular dichroïsm **(Fig. S2C**).

### Enzymatic Characterization

OPHC2 enzyme was initially characterized for its ability to hydrolyze OPs in crude extracts [19]. We have determined the enzyme kinetic parameters for several insecticides: ethyl/methyl-paraoxon (**Fig. S1I–II**), ethyl/methyl-parathion (**Fig. 1SIII–IV**) and malathion (**Fig. 1SV**) (**Table 2**). Methyl-paraoxon (k_cat_/K_M_ = 1.48(±0.34)×10^3^ M^−1^s^−1^) and methyl-parathion (k_cat_/K_M_ = 2.68(±0.73)×10^3^ M^−1^s^−1^) are the best substrates. As previously observed in PLLs [28], the K_M_ for methyl-parathion is better than for methyl-paraoxon. Ethyl-paraoxon (k_cat_/K_M_ = 13.3±9.2 M^−1^s^−1^), and malathion (specific activity = 329±82 µmol.mol^−1^.s^−1^) are poor substrates for OPHC2, whereas CMP-coumarin (**Fig. 1B**) is hydrolyzed with significant rates (k_cat_/K_M_ = 2.96(±0.48)×10^3^ M^−1^s^−1^)_._ Overall, OPHC2 exhibits rather low organophosphate hydrolase activity (k_cat_/K_M_∼10^3^ M^−1^s^−1^) as compared to other enzymes such as PTEs, mainly because of a low catalytic rate (k_cat_∼10^−1^ s^−1^).

**Table 2:** Enzymatic characterisation of OPHC2 enzyme.

	Substrates	k_cat_ (s^−1^)	K_M_ (µM)	k_cat_/K_M_ (M^−1^s^−1^)
**Phosphoesters**	**Ethyl-paraoxon (I)**	4.05(±0.01)×10^−3^	94±19	1.33(±0.92)×10^1^
	**Methyl-paraoxon (II)**	3.87(±0.29)×10^−1^	261±56	1.48(±0.34)×10^3^
	**Ethyl-parathion (III)**	ND	ND	ND
	**Methyl-parathion (IV)**	5.71(±0.33)×10^−2^	21±6	2.68(±0.73)×10^3^
	**Malathion (V)**	ND	ND	VLH
	**CMP-coumarin (VI)**	3.38(±0.19)×10^−1^	114±17	2.96(±0.48)×10^3^
**Esters**	**Phenyl-acetate (VII)**	9.03(±1.26)×10^−2^	1620±563	5.56(±2.08)×10^1^
	***p*** **NP-Acetate (VIII)**	ND	ND	2.17(±0.08)×10^1^
	***p*** **NP-Decanoate (IX)**	3.22(±0.25)×10^−2^	138±48	2.33(±0.83)×10^2^
**Lactones**	**AHLs (X–XV)**	ND	ND	ND
	**oxo-lactones (XVI–XX)**	ND	ND	ND
	**Di-hydrocoumarin (XXI)**	2.39±0.20	403±100	5.93(±1.55)×10^3^

Roman numbers correspond to the related chemical structure of the substrate presented in **Figure S1**. Data obtained with cobalt as cofactor. ND corresponds to not determined values because of no or too low catalytic rate. VLH correspond to Very Low Hydrolysis observed without the possibility to record a value.

Concomitantly to the phosphotriesterase activity, esterase or lactonase activities are systematically observed in other enzyme superfamilies such as paraoxonases or PLLs [12], [13]. We have thus characterized OPHC2’s activity against various esters (**Fig. 1C**) and lactones (**Fig. 1D–F**) (**Table 2**). OPHC2 hydrolyzes phenyl-acetate, *p*NP-acetate and *p*NP-decanoate with low catalytic efficiencies (k_cat_/K_M_<10^2^ M^−1^s^−1^) because of low catalytic rates (k_cat_∼10^−2^ s^−1^). The best ester substrate for OPHC2 is *p*NP-decanoate (k_cat_/K_M_ = 2.33(±0.83)×10^2^ M^−1^s^−1^). Concerning lactones, no hydrolysis of AHLs (**Fig. 1D**) or oxo-lactones (**Fig. 1EF**) could be detected, even at high enzyme concentration (*i.e.* 250 µg/mL). However, interestingly, the best substrate for OPHC2 of all tested molecules is the lactone dihydrocoumarin, with a catalytic efficiency of 5.93(±1.55)×10^3^ M^−1^s^−1^.

### Structural Characterization

#### X-Ray structure of OPHC2

**Figure 3:**
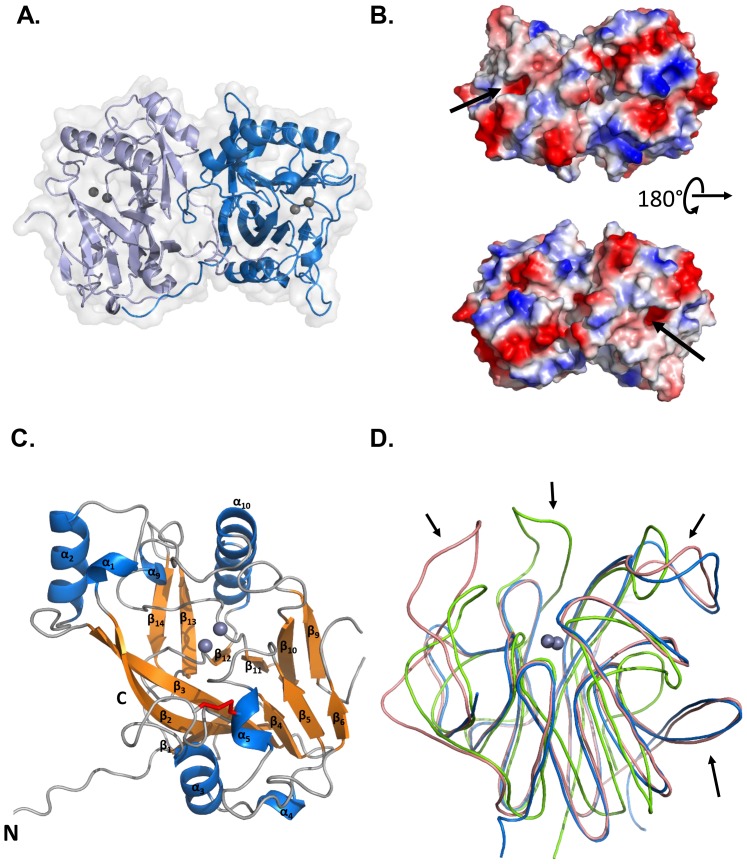
Structure of OPHC2. A. General representation of the OPHC2 dimer. Monomers are colored in light and dark blue. The two metals of the active site are represented as grey spheres. Enzyme surface is also represented. B. Electrostatic surface of OPHC2 dimer. Positive and negative potentials are colored in blue and red, respectively. The active sites are indicated by black arrows. C. Cartoon representation of an OPHC2 monomer with α helices colored in blue, β sheets in orange and loops in grey. The secondary structures are numbered from the alignment present in Fig. 2B. The bimetallic centre is shown as two grey spheres and the disulphide bridge is shown as red sticks. N- and C-terminal extremities are also indicated. D. Structural comparison of OPHC2 (in blue) with MPH (in salmon) and AiiA (in green). Major differences concern loops sizes and conformations.

The structure of OPHC2 was solved at 2.1 Å resolution (**Table 1**) and reveals that OPHC2 forms a homodimer in the crystal. The dimer interface consists in a large, mainly hydrophobic, contact area between the two monomers (2453.2 Å^2^) (**Fig. 3A**), which is bigger than that of MPH (2243.9 Å^2^). The dimer is reinforced by the interaction of the N-terminal extremities of the chain that contact the second monomer (**Fig. 3A**). Both monomers interact intensively, performing 29 hydrogen bonds and 15 salt bridges, involving 61 residues (256 atoms). In comparison, MPH monomers perform 45 hydrogen bonds and 8 salt bridges. OPHC2 exhibits a very charged surface (**Fig. 3B** **)** with several surface salt bridges (****) that stabilize, for example, the protein extremities (**Fig. S3B**). Finally, the presence of a disulphide bridge (Cys110-Cys146), absent from MPH, may contribute to the higher thermostability of OPHC2 [44].

The monomer of OPHC2 is roughly globular with overall dimensions of approximately 44 Å×50 Å×37 Å. As for MPH, its structure could be described as an αβ/βα sandwich topology, typical of the metallo-β-lactamase superfamily [42]. Two mixed twisted β-sheets, each composed of six strands, are flanked by seven α-helices exposed to the solvent (**Fig. 3C**). The catalytic center is located between the two β-sheets and surrounded by the connecting αβ-loops. In OPHC2 structure, residues 168 to 210 are absent from the electron density maps and therefore have not been modelled. A tentative model of these missing residues based on MPH structure yielded a model that is unfortunately not compatible with OPHC2’s crystal packing (**Fig. S4**).

The superposition of OPHC2 with MPH and AiiA yields RMSD values for α-carbon atoms of 0.74 Å (over 213 residues) and 2.41 Å (over 105 residues), respectively. While OPHC2 and MPH structures are very similar, major structural differences are visible between OPHC2 and AiiA. These differences mainly concern the loops size and conformations (**Fig. 3D** **)**.

#### Active site description

**Figure 4:**
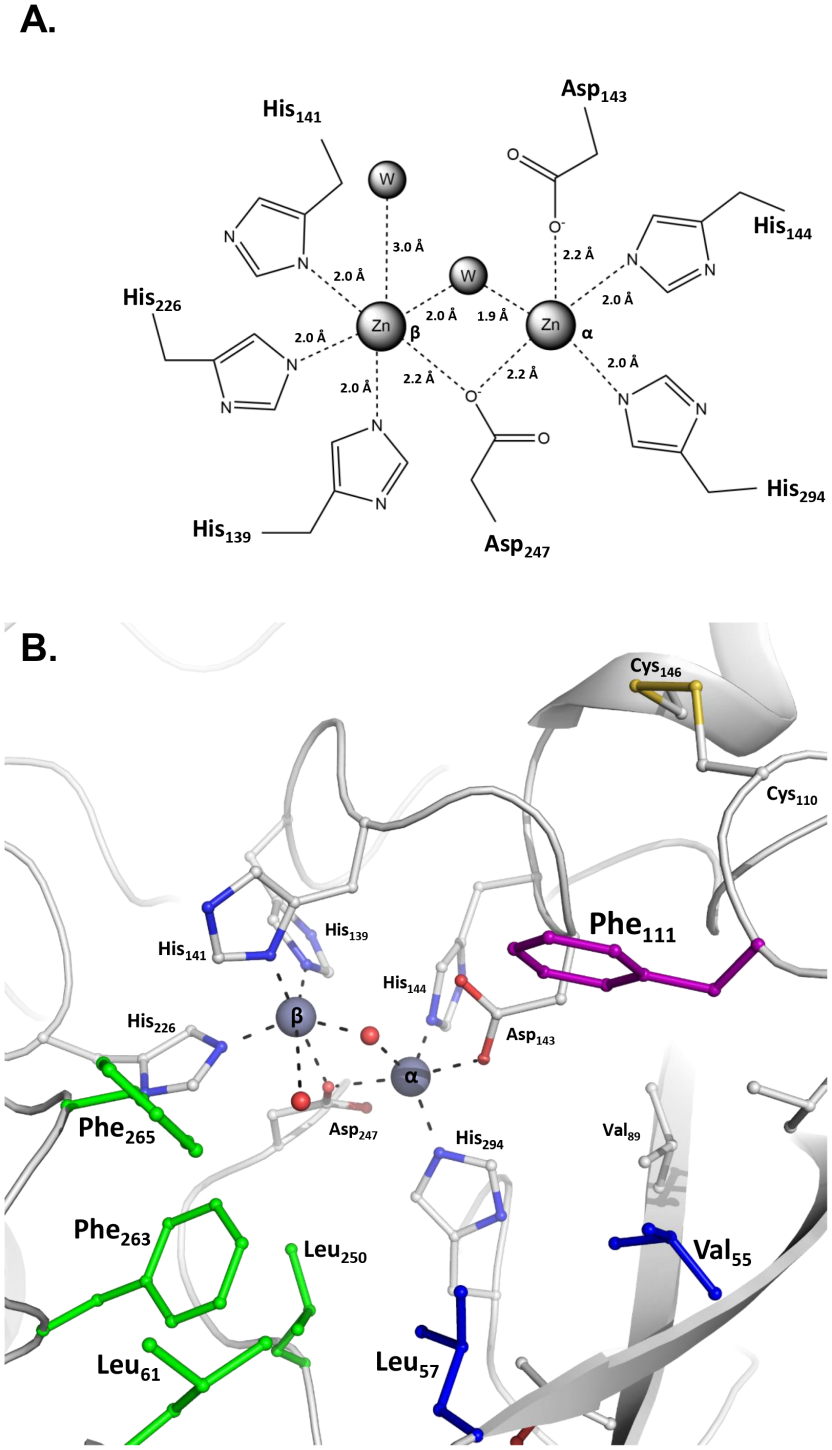
Active site of OPHC2. **A.** Schematic representation of the bi-metallic centre of OPHC2. The two metals and the two water molecules are represented as grey balls. **B.** Three dimensional representation of the OPHC2 active site cavity. Residues are shown as sticks. The metal coordination sphere is shown as dashed lines. The disulphide bridge between Cys110 and Cys146 is colored in yellow. The leaving group subsite of OPHC2 is colored in purple, and the two side pockets are colored in green and blue.

The active site of OPHC2 consists of a cavity with two metal cations: one buried (α metal) and one more solvent exposed (β metal). The α metal is coordinated by His294, His144, Asp143 and the Asp247, the latter coordinates also the β metal together with His226, His139, His141 and a water molecule (**Fig. 4A**). Both metals are bridged by the putative catalytic water molecule. The chemical nature of metal cations was investigated using anomalous X-ray data collection at the Zn-K edge (1.2822 Å) and under (1.2835 Å) (**Table S2**). The presence of two peaks for each metals in Bijvoet difference Fourier maps at the Zn-K edge (18.5 and 14.7 σ in height) and their drop in the maps calculated from data collected under the Zn-edge (8.9 and 11.6 σ) clearly indicates the presence of zinc cations in the active site, but not only (**Table S2**). Indeed, the residual fluorescence observed under the Zn absorption edge may be due to the presence of iron or cobalt, as observed in the X-ray fluorescence spectrum (**Fig. S5**). Therefore, the active site of OPHC2 contains, at both α and β positions, a mixture of zinc and possibly cobalt and/or iron.

The substrate binding pocket is mainly composed of hydrophobic residues. It can be subdivided, based on the MPH structure [45], into three subsites: the leaving group pocket (Phe111, Met188 and Trp172), a first specificity subsite (Leu250, Leu61, Phe263 and Phe265) and a second specificity subsite (Val55 and Leu67) (**Fig. 4B** and **Fig. S6)**. Residues Met188 and Trp172 that belong to the leaving group subsite in MPH, belong to the disordered protein fragment that has not been modelled. Residues Cys110 and Cys146 that form a disulphide bridge comprise second shell active site residues (**Fig. 4B**). It covalently bridges helix α_5_ and loop β_4_α_3_, and may thus rigidify the active site, especially Phe111, a residue possibly involved in the leaving group subsite.

## Discussion

### OPHC2 is a Dimeric, Thermostable Enzyme

OPHC2 enzyme, as observed for MPH [22], crystallizes as a homodimer, and biochemical evidences suggests that this dimer exists in solution. This homodimer exhibits (i) a very important surface interaction, (ii) a high number of intermolecular hydrogen bonds and salt bridges. The higher thermal stability of OPHC2, as compared to its closest known homologue MPH, may stem from a high dimer interface area and an intramolecular disulfide bridge. Moreover, the structure reveals several ionic bridges at the protein surface, a feature commonly observed in thermostable enzymes [46], [47] and usually linked to thermal stability. Altogether, these structural determinants may contribute to the enzyme thermal stability (T_m_ of 97.8±3.2°C). Because of this very high stability, OPHC2 can be purified by a fast and easy procedure: a heating step followed by ammonium sulfate precipitation, and a polishing step by gel filtration. Moreover, being a thermostable enzyme [47], OPHC2 is expected to exhibit high stability toward various chemicals like organic solvent, and resist to long-term storage. These properties and its measured organophosphate-degrading ability make OPHC2 an interesting candidate for developing an efficient OP biodecontaminant by protein engineering.

### OPHC2 Exhibits Esterase and Phosphotriesterase Activities

We here show that OPHC2 hydrolyzes a broad range of esters, from phosphotriesters to the lactone dihydrocoumarin. Being isolated as a phosphotriesterase [19], we show that the enzyme hydrolyzes various insecticides and a nerve agent analogue with relatively low catalytic efficiencies. OPHC2 exhibits clear preference for small substituents (*e.g.* OPHC2 processes methyl-parathion better than ethyl-parathion). Notably, OPHC2 is a less efficient phosphotriesterase than its closest homologue MPH (*e.g*., against methyl parathion), catalytic efficiencies of OPHC2 and MPH are ∼10^3^ M^−1^s^−1^ and ∼10^5^ M^−1^s^−1^ [22], respectively. This lower activity seems to reside in low k_cat_ values of OPHC2 for phosphotriesters. While structures of MPH and OPHC2 are overall similar (RMSD = 0.74 Å), some differences are observed in active site residues, loops lengths, and second shell residues. Laboratory evolution studies have repeatedly shown that substrate preferences are mediated, at least partly, by length and conformation of active site surrounding loops [16], [48]–[50]. OPHC2’s active site comprises a highly hydrophobic substrate binding pocket which thus seems well adapted for the accommodation of the hydrophobic molecules that comprise OPs. The sub-sites architecture of OPHC2 was established on the basis of that of MPH [7], [22] (see **Fig. S6**). Comparison of both active site highlights amino acids differences in the leaving group pocket (*e.g.* Met188_OPHC2_ instead of Phe196_MPH_), in the side pockets (*e.g* Phe265_OPHC2_ instead of Leu273_OPHC2_ and Leu61_OPHC2_ instead of Arg72_MPH,_ and the presence of an additional Phe263_OPHC2_ in the binding pocket), as well as the floppy region 168–210. All these differences probably account for the differences in substrate specificity and catalytic efficiencies of both proteins. These residues may thus represent interesting targets for mutational studies, with the aim of increasing the phosphotriesterase activity and widening the specificity spectrum of OPHC2.

The similarities between catalytic centers of OPHC2 and others OPHs, however, suggest a similar enzymatic chemistry. The catalytic center is composed of two metals bridging an activated water molecule as observed in MPH and AiiA structures [22], [51], but also other esterases such as *Sso*Pox or *Sis*Lac [17], [18]. Consequently, the hydrolysis mechanism of OPHC2 might be common to these related enzymes.

Additionally, OPHC2 exhibits lactonase activity. Amongst the 12 tested lactones, OPHC2 processes, however, only dihydrocoumarin. Despite the absence of the conserved Tyr residue, characteristic of lactonases in the metallo-β-lactamase superfamily [52] and in the PLLs [16], [17], [53], the catalysis of dihydrocoumarin is significant (k_cat_/K_M_ = 5.93×10^3^ M^−1^s^−1^). A low lactonase activity, interestingly, has been recorded for many phosphotriesterases, the molecular promiscuity between both activities being hypothesized to stem from a molecular overlap between substrate binding of the phosphotriesters and the transition state of the lactone hydrolysis [13].

Finally, we here show that OPHC2 exhibits relatively low catalytic efficiencies against the range of tested substrates (∼10^3^ s^−1^M^−1^ against the best substrates). The average catalytic efficiency of enzymes being ∼10^5^ s^−1^M^−1^ [54], this work suggests that OPHC2 natively processes a substrate, yet unknown, that is different in chemical nature from the tested molecules of this study. OPHC2 may therefore have a different biological function than being a lactonase, a phosphotriesterase or an esterase.

## Supporting Information

Figure S1 **Chemical structure of phosphoesters (I-VI), esters (VII-IX) and lactones (X-XXI).**
(DOCX)

Figure S2 **Biochemical characterization of OPHC2.**
(DOCX)

Figure S3 **Surface salt bridges of OPHC2.**
(DOCX)

Figure S4 **Modelization of the missing part of OPHC2.**
(DOCX)

Figure S5 **X-ray fluorescence spectrum of OPHC2 crystal.**
(DOCX)

Figure S6 **Substrate specificity and subsites comparison between MPH (A.) and OPHC2 (B.).**
(DOCX)

Table S1 **Protein sequence identity between OPHC2, MPH and AiiA-B.**
(DOCX)

Table S2 **Anomalous X-ray data collection.**
(DOCX)
