# Supplementary material for: Structural and Enzymatic Characterization of the Phosphotriesterase OPHC2 from Pseudomonas pseudoalcaligenes
Source: PLoS One. 2013 Nov 4;8(11):e77995. doi: 10.1371/journal.pone.0077995 (PMC3817169; doi:10.1371/journal.pone.0077995)
Supplement: Figure S1 — Chemical structure of phosphoesters (I-VI), esters (VII-IX) and lactones (X-XXI). (DOCX) [file pone.0077995.s001.docx]

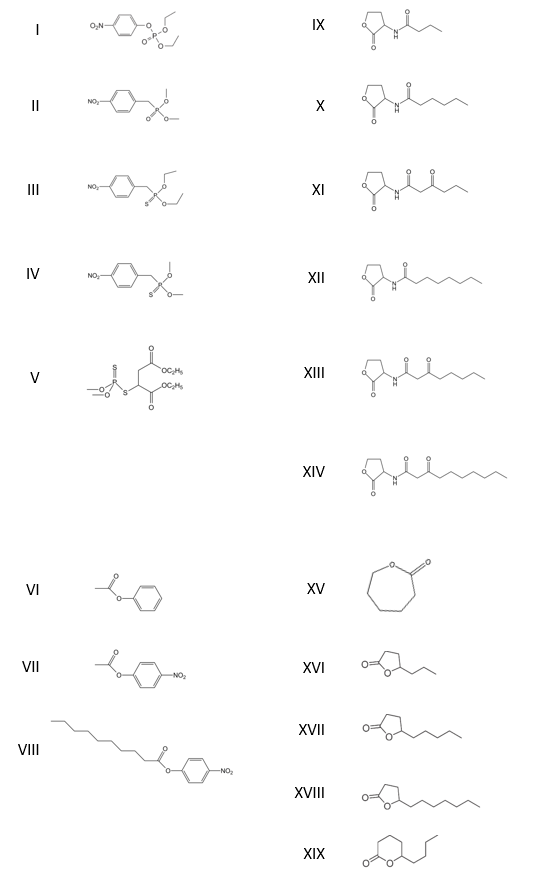


**Figure S1: Chemical structure of phosphoesters (I-VI), esters (VII-IX) and lactones (X-XXI)**

Chemical structure of ethyl-paraoxon (I), methyl-paraoxon (II), ethyl-parathion (III), methyl-parathion (IV), malathion (V), phenyl-acetate (VI), *p*NP-acetate (VII), *p*NP-decanoate (VIII), .C4-AHL (IX), C6-AHL (X), 3-oxo-C6-AHL (XI), C8-AHL (XII), 3-oxo-C8-AHL (XIII), 3-oxo-C12-AHL (XIV), ε-caprolactone (XV), γ-heptanolide (XVI), Nonanoic-γ-lactone (XVII), Undecanoic-γ-lactone (XVIII) and Nonanoic-δ-lactone (XIX).
