# Supplementary material for: Structural and Enzymatic Characterization of the Phosphotriesterase OPHC2 from Pseudomonas pseudoalcaligenes
Source: PLoS One. 2013 Nov 4;8(11):e77995. doi: 10.1371/journal.pone.0077995 (PMC3817169; doi:10.1371/journal.pone.0077995)
Supplement: Figure S2 — Biochemical characterization of OPHC2. (DOCX) [file pone.0077995.s002.docx]

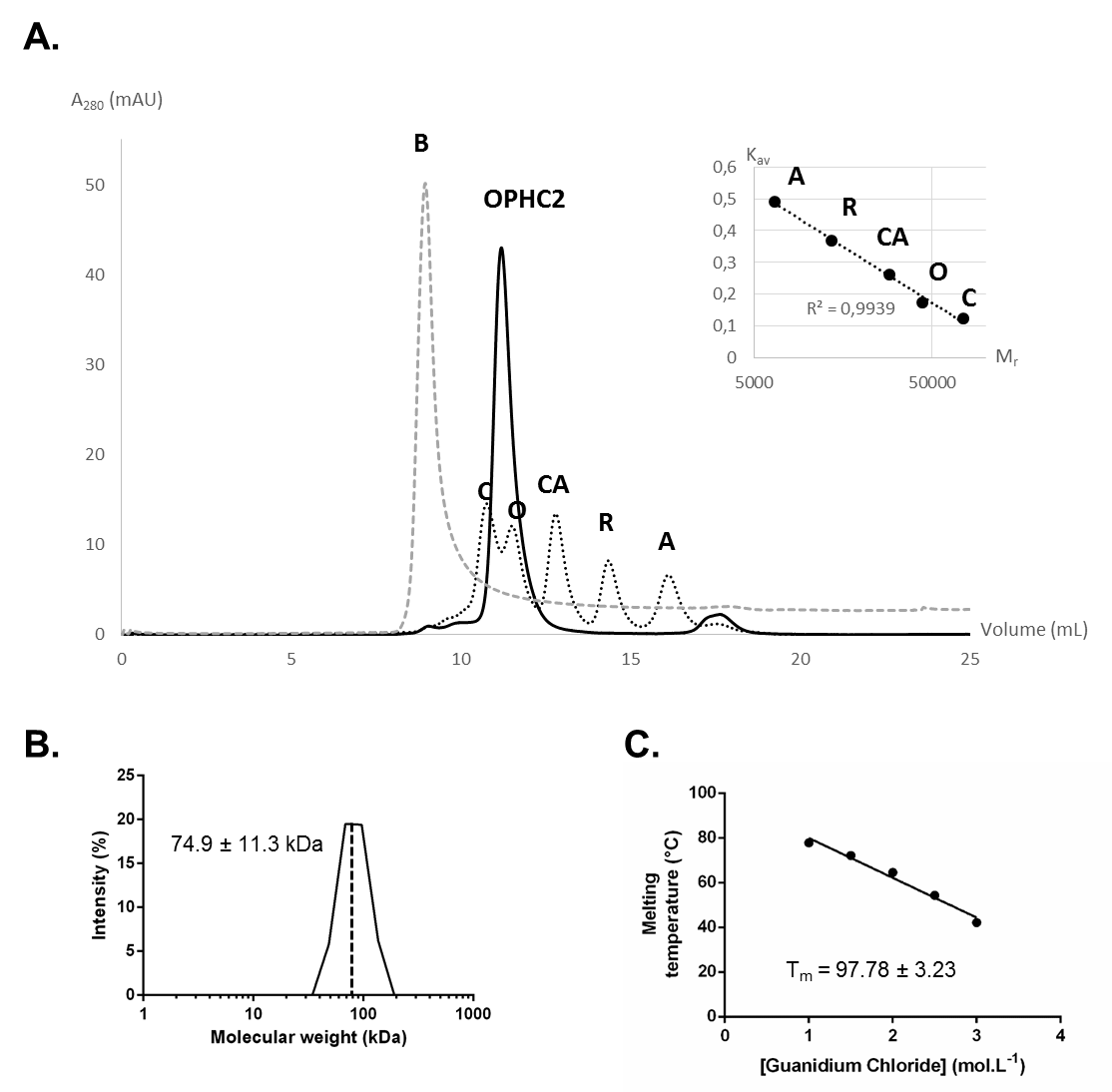


**Figure S2: Biochemical characterization of OPHC2**

**A.**Oligomerization state analysis of OPHC2 by exclusion size chromatography. Black line corresponds to the elution profile of 145 µg of OPHC2 on the S75 10/300 GL chromatography column. Black dashed lines correspond to the elution profile of the gel filtration low molecular weight calibration kit (GE-Healthcare): Conalbumin (C), Ovalbumin (O), Carbonic Anhydrase (CA), Ribonuclease A (R), Aprotinin (A). Grey dashed lines correspond to the elution profile of the Blue Dextran 2000 (B) used to obtain the void volume of the column. The apparent molecular weight of OPHC2 was inferred from the calibration curve of the *K_av_* function of the molecular weight (M_r_). *K_av_* was calculated from the formula:

$K_{av}=\frac{V_{e}-V_{0}}{V_{c}- V_{0}}$ , where *V_e_* is the elution volume of each protein, *V_0_* is the void volume of the column and *V_c_* is the geometric volume of the column. **B.** Oligomerisation state analysis of OPHC2 by DLS experiment. DLS profile of 30 µL OPHC2 enzyme at 5 mg.ml^-1^ averaged on 5 experiments. **C.**Melting temperature (T_m_) determination of OPHC2 enzyme. Linear regression of the OPHC2 T_m_ *versus* guanidinium chloride concentration which allow to extrapolate the T_m_ of the protein at 97.8 ± 3.2 °C at the y-intercept.
