# Supplementary material for: Structural and Enzymatic Characterization of the Phosphotriesterase OPHC2 from Pseudomonas pseudoalcaligenes
Source: PLoS One. 2013 Nov 4;8(11):e77995. doi: 10.1371/journal.pone.0077995 (PMC3817169; doi:10.1371/journal.pone.0077995)
Supplement: Figure S3 — Surface salt bridges of OPHC2. (DOCX) [file pone.0077995.s003.docx]

**
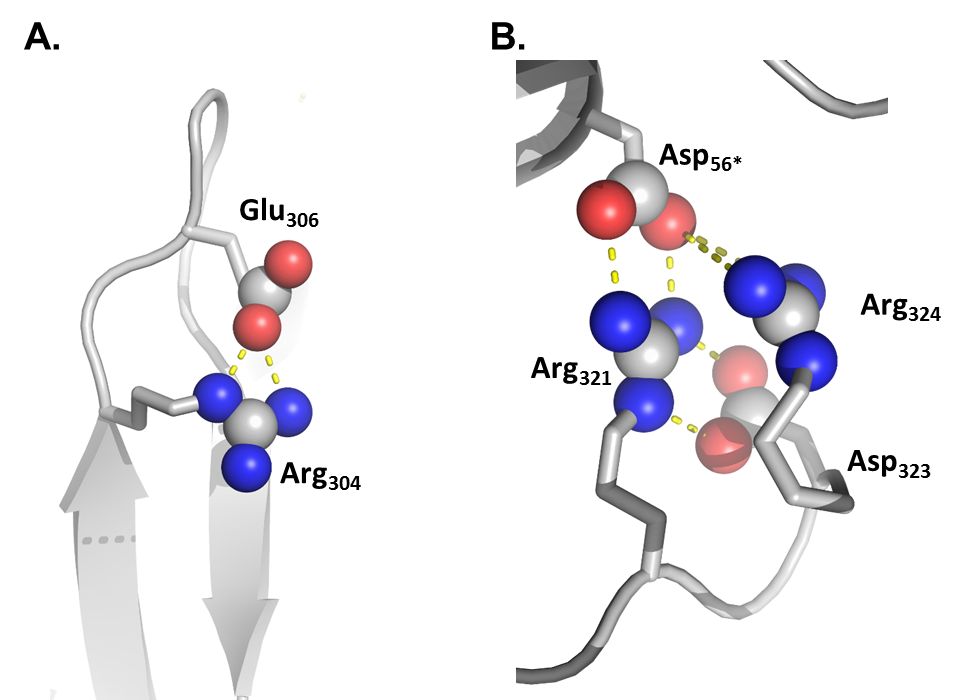
**

**Figure S3: Surface salt bridges of OPHC2**

Some surface salt bridges in OPHC2 structure. Main chain and carbon atoms are colored in grey. Oxygen and nitrogen atoms are represented as red and blue spheres respectively. Interactions between complementary charges are indicated by yellow dashes. **A.** Typical salt bridge at the surface of the OPHC2 structure. **B.** Complex salt bridge network linking the extremity of one monomer to its associated monomer (indicated by a star).
