# Supplementary material for: Structural and Enzymatic Characterization of the Phosphotriesterase OPHC2 from Pseudomonas pseudoalcaligenes
Source: PLoS One. 2013 Nov 4;8(11):e77995. doi: 10.1371/journal.pone.0077995 (PMC3817169; doi:10.1371/journal.pone.0077995)
Supplement: Figure S4 — Modelization of the missing part of OPHC2. (DOCX) [file pone.0077995.s004.docx]

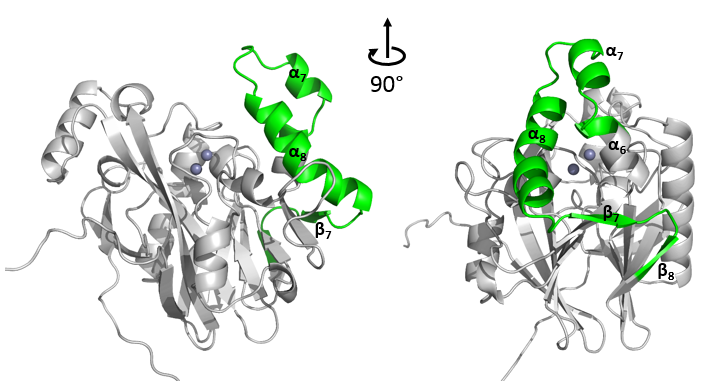


**Figure S4: Modelization of the missing part of OPHC2**

Cartoon representation of the OPHC2. The part missing from the crystallographic structure is colored in green and secondary structures are numbered as in the alignment of **Fig. 2B**. This part was modelled using *MODELLER* and MPH structure as a model. The two metals are shown as spheres. The quality of the structure was validated using Rampage software from the CCP4 suite (Ramachandran favoured regions, 93.6 %; Allowed region, 4.4 %; generously allowed, 2.0 %).
