# Supplementary material for: Structural and Enzymatic Characterization of the Phosphotriesterase OPHC2 from Pseudomonas pseudoalcaligenes
Source: PLoS One. 2013 Nov 4;8(11):e77995. doi: 10.1371/journal.pone.0077995 (PMC3817169; doi:10.1371/journal.pone.0077995)
Supplement: Figure S5 — X-ray fluorescence spectrum of OPHC2 crystal. (DOCX) [file pone.0077995.s005.docx]

**
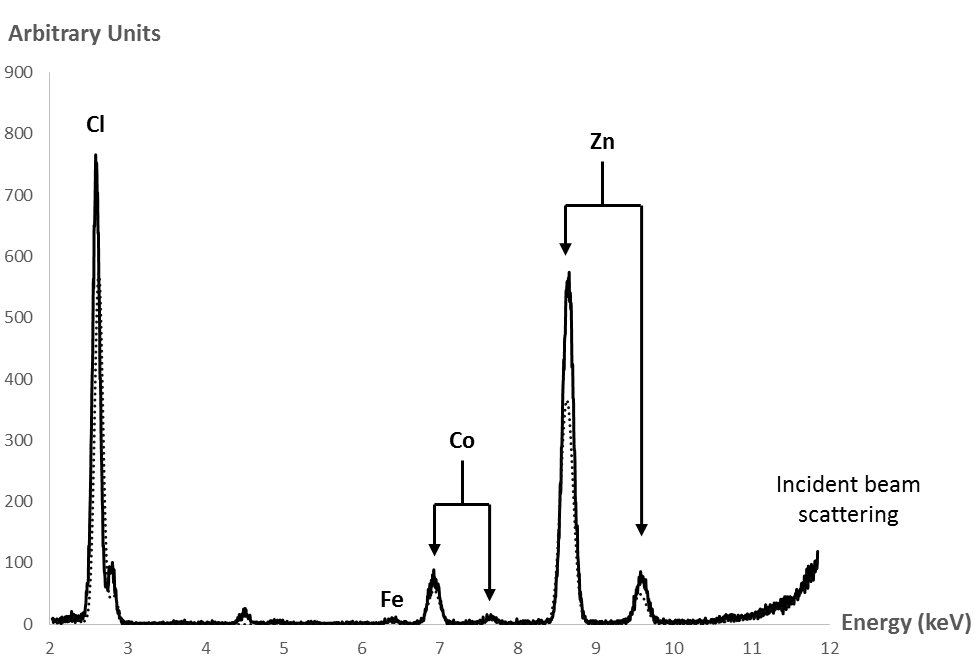
**

**Figure S5: X-ray fluorescence spectrum of OPHC2 crystal**

X-ray fluorescence spectrum of OPHC2 crystals. Data have been analysed using *PyMCA* software [[53](#_ENREF_53)] revealing the presence of chloride, cobalt, zinc and iron. Doubled arrows indicate the characteristic Kα and Kβ edges of compounds.
