# Supplementary material for: Structural and Enzymatic Characterization of the Phosphotriesterase OPHC2 from Pseudomonas pseudoalcaligenes
Source: PLoS One. 2013 Nov 4;8(11):e77995. doi: 10.1371/journal.pone.0077995 (PMC3817169; doi:10.1371/journal.pone.0077995)
Supplement: Figure S6 — Substrate specificity and subsites comparison between MPH (A.) and OPHC2 (B.). (DOCX) [file pone.0077995.s006.docx]

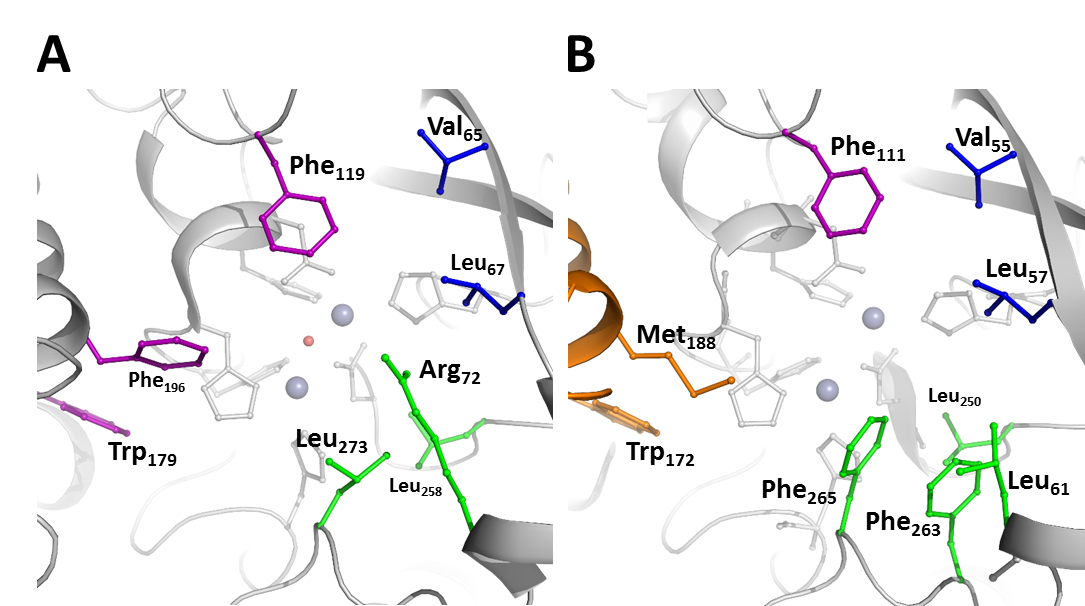


**Figure S6: Substrate specificity and subsites comparison between MPH (A.) and OPHC2 (B.)**

Leaving group pocket residues are colored in purple, side pockets are colored in green and blue. Residues absent from the crystallographic structure but modelled are colored in orange.
