# Supplementary material for: Structural and Enzymatic Characterization of the Phosphotriesterase OPHC2 from Pseudomonas pseudoalcaligenes
Source: PLoS One. 2013 Nov 4;8(11):e77995. doi: 10.1371/journal.pone.0077995 (PMC3817169; doi:10.1371/journal.pone.0077995)
Supplement: Table S1 — Protein sequence identity between OPHC2, MPH and AiiA-B. (DOCX) [file pone.0077995.s007.docx]

**Table S1: Protein sequence identity between OPHC2, MPH and AiiA-B**

|  | OPHC2-1 | OPHC2-2 | MPH | AiiA | AiiB |
| --- | --- | --- | --- | --- | --- |
| OPHC2-1 | - | 98 % | 45 % | 18 % | 14 % |
| OPHC2-2 | - | - | 44 % | 17 % | 14 % |
| MPH | - | - | - | 18 % | 9 % |
| AiiA | - | - | - | - | 22 % |
| AiiB | - | - | - | - | - |

OPHC2-1 originates from *P. pseudoalcaligenes*. OPHC2-2 originates from *Stenotrophomonas* sp. SMSP-1. MPH originates from *Stenotrophomonas* sp. Dsp-4. AiiA originates from *B. thuringiensis*. AiiB originates from *A. fabrum* str. C58. Identities were obtained from a sequence alignment preformed using the ClustalW server.
