# Supplementary material for: Structural and Enzymatic Characterization of the Phosphotriesterase OPHC2 from Pseudomonas pseudoalcaligenes
Source: PLoS One. 2013 Nov 4;8(11):e77995. doi: 10.1371/journal.pone.0077995 (PMC3817169; doi:10.1371/journal.pone.0077995)
Supplement: Table S2 — Anomalous X-ray data collection. (DOCX) [file pone.0077995.s008.docx]

**Table S2: Anomalous X-ray data collection**

| Data collection | | |
| --- | --- | --- |
| Dataset | Zn-K edge high | Zn-K edge low |
| Beamline | ID29 | ID29 |
| Wavelength (Å) | 1.2822 Å | 1.2835 Å |
| Detector | PILATUS 6M | PILATUS 6M |
| Oscillation (°) | 0.1 | 0.1 |
| Number of frames | 3600 | 3600 |
| Resolution (Å) (last bin) | 3.1 (3.2-3.1) | 2.6 (2.7-2.6) |
| Space group | C2 | C2 |
| Unit-cell parameters (Å) | a = 111.7, b = 63.8, c = 222.04, β = 101.5 | a = 111.2, b = 63.7, c = 222.0, β = 101.7 |
| No. of observed reflections (last bin) | 181006 (17769) | 312228 (34740) |
| No. of unique reflections (last bin) | 54065 (5015) | 91173 (9834) |
| Completeness (%)(last bin) | 99.2 (99.8) | 99.3 (99.7) |
| R_meas_ (%) (last bin) | 10.8 (57.5) | 9.5 (54.5) |
| I/σ(I) (last bin) | 10.83 (3.1) | 11.60 (3.40) |
| Redundancy (last bin) | 3.35 (3.54) | 3.42 (3.53) |
| Mosaicity (°) | 0.155 | 0.120 |
| Anomalous peak (σ)^#^ | α metal = 18.5  β metal = 14.7 | α metal = 8.9  β metal = 11.6 |
| ^#^ is the height of the anomalous peak (in σ units) in the Bijvoet difference Fourier map at the location of the metal cations in the structure. The anomalous signal decline at lower energy, showing that the binding pocket is occupied by zinc ions, but not only. | | |
